# Elitist Binary Wolf Search Algorithm for Heuristic Feature Selection in High-Dimensional Bioinformatics Datasets

**DOI:** 10.1038/s41598-017-04037-5

**Published:** 2017-06-28

**Authors:** Jinyan Li, Simon Fong, Raymond K. Wong, Richard Millham, Kelvin K. L. Wong

**Affiliations:** 1 Department of Computer and Information Science, University of Macau, Macau SAR, China; 2 School of Computer Science and Engineering, University of New South Wales, New South Wales, Australia; 3 Department of Information Technology, Durban University of Technology, Durban, South Africa; 4 School of Medicine, Western Sydney University, New South Wales, Australia; 5 Centre for Biomedical Engineering, School of Electrical & Electronic Engineering, University of Adelaide, Adelaide, Australia

## Abstract

Due to the high-dimensional characteristics of dataset, we propose a new method based on the Wolf Search Algorithm (WSA) for optimising the feature selection problem. The proposed approach uses the natural strategy established by Charles Darwin; that is, ‘*It is not the strongest of the species that survives*, *but the most adaptable*’. This means that in the evolution of a swarm, the elitists are motivated to quickly obtain more and better resources. The memory function helps the proposed method to avoid repeat searches for the worst position in order to enhance the effectiveness of the search, while the binary strategy simplifies the feature selection problem into a similar problem of function optimisation. Furthermore, the wrapper strategy gathers these strengthened wolves with the classifier of extreme learning machine to find a sub-dataset with a reasonable number of features that offers the maximum correctness of global classification models. The experimental results from the six public high-dimensional bioinformatics datasets tested demonstrate that the proposed method can best some of the conventional feature selection methods up to 29% in classification accuracy, and outperform previous WSAs by up to 99.81% in computational time.

## Introduction

Data size can be measured using two aspects: the number of features and the number of samples. A large number of features can cause serious problems, such as the Curse of Dimensionality^1^, and the dimensions of feature spaces should not be too high, due to an empirical axiom in machine learning^2^. In recent years, dataset sizes have skyrocketed in my many bioinformatics applications^3^, such as text mining, image processing, gene chromosome engineering and biological engineering. Given that it is difficult to determine whether the extracted features have enough information, to improve data identification, feature extraction is used to extract as many features as possible. This operation increases dataset dimensions with some invalid, nonrelated and redundant features. These massive datasets can then lead to serious problems for and challenges to the performance and measurability of machine learning algorithms.

Feature selection is a commonly and effectively used method of feature dimension reduction in selecting a suitable low-dimensional sub-dataset from an initial high-dimensional dataset^4, 5^. In machine learning, feature selection can be expressed as follows. Given machine learning algorithm *A*, dataset *D* and *d* as taken from a categorised sample space with the features *F*_*1*_, *F*_*2*_, *F*_*3*_ *, …*, *F*_*n*_, there is an optimal sub-dataset *D*_*x*_ that can offer best evolution indicator *E* = *E(A*, *D)*. The meanings of feature selection are as follows. First, reduce the negative effects of invalid, nonrelated and redundant features to improve accuracy. Second, use a low-dimensional dataset to replace a high-dimensional dataset, which decreases the computational cost and improves adaptability. Feature selection can be considered an optimisation problem. There are *N*! / [(*N* − *n*)! × *n*!] candidate solutions to select n sub-features from *N* features in the search space. For instance, if *n* = 10 and *N* = 100, the number of candidate solutions is 1.731e + 13. Furthermore, the number of n is an undefined value, thus we must find the best combination of features at the best length. In addition, previous researchers have proven that searching for the best sub-dataset is an NP problem^6^, which means that there are no other methods to guarantee that the optimal solution must be found, other than the exhaustive method. However, the dimensions increase as the dataset grows, thus the huge computational cost of the exhaustive method is not practical for real applications. Researchers are now studying swarm intelligence algorithms, which are heuristic methods, to find an optimal or second-best solution.

There are several versions of heuristic algorithms for feature selection. Kennedy and Russell created a well-known method called Binary Particle Swarm Optimisation (BPSO)^7^, and Russell extended the BPSO research to feature selection^8^, which changed the traditional feature selection pattern to processing a binary optimisation problem. Recently, Tang proposed the Wolf Search Algorithm (WSA), inspired by the behaviour displayed by wolves while hunting^9, 10^. Its jump mechanism helps the wolves effectively avoid falling into a local optimum. In previous research, WSAs have adopted wrapper strategies with different traditional classifiers, significantly exceeding some well-known swarm-based feature selection methods in classification performances^11, 12^. However, the high computational time cost is the problem requiring optimisation. The new binary version of the WSA for feature selection proposed in this paper is called the Elitist Binary Wolf Search Algorithm (EBWSA). The size of the search space is 2 × *N*, where *N* denotes the number of features. In such cases, the program avoids simultaneously considering the best length and combination of sub-features from the 2*N* − 1 possible candidate solutions. Moreover, the elitist mechanism drives the better wolves to lead the whole population toward a better solution within a shorter computational time. This type of competitive strategy also accelerates the elimination and rebirth of the worst wolves. In addition, the memory function increases the effective and positive search by considering the previous worst positions in the limited memory, because an overly long memory length increases the time cost of input/output (I/O). Compared with other non-iterative traditional methods, such as BPSOs and WSAs, the proposed method could improve the accuracy of classification models with high computational and convergence speeds.

As mentioned above, the classical definition of feature selection is selecting a sub-dataset *d* with *f* features from the primary dataset *D* with *F* features, *f* ≤ *F*, such that *d* exhibits optimal performance in all of the sub-datasets with f features from the primary dataset^6^. Based on the basic framework of feature selection, four necessary steps are proposed for the feature selection procedur^13^: subset generation, subset evaluation, stop criteria and results validation. Subset generation is a search process that uses corresponding strategies to select preselected sub-datasets. The evaluated metrics of each preselected sub-dataset must then be compared with the same metrics for the current best sub-dataset. If a preselected sub-dataset is better than the current best, the former replaces the latter. Subset generation and evaluation cycle until the stop criteria are met. Finally, the selected sub-dataset is validated to build the model.

Therefore, during the feature selection process, in addition to finding a suitable algorithm to select an optimal sub-dataset with distinguished selected features in the shortest time, cost is very important, and the evaluation metrics are essential in estimating whether the selected features are optimal. Thus, feature selection approaches can be divided into two types: those that function according to the strategy for searching subsets, and those that do so according to the evaluation standard for features.

There are three strategies for searching subsets: global optimisation^14^, heuristic^15^ and random^2^. The exhaustion method traverses all of the feature combinations in a feature space, making it one of the most straightforward approaches. However, due to the computational complexity of O(2^*N*^), the exhaustion method is infeasible when the objective is a high-dimensional dataset. The Branch and Bound algorithm^16, 17^ is the only method that uses a global optimisation search strategy to obtain an optimal solution. Compared with exhaustion, Branch and Bound reduces the time cost but uses monotonic evaluation functions, which can be difficult to design. Moreover, its efficiency is still significantly lower when tackling high-dimensional problems. The heuristic searching strategy is an approximation algorithm that adopts a compromise strategy between searching performance and computational complexity. It generally obtains a solution that is approximated to the optimal, and its computation complexity is equal to or smaller than O(*N* ^2^). Sequential forward selection (SFS) and sequential backward selection (SBS)^18^ are two of the most typical heuristic searching strategies. SFS uses a top-down searching strategy in which the initial selected feature set is empty, and a feature is added to the set in each searching time until the set reaches the requirement. Generalised SFS is the accelerated version of the strategy. SBS is the opposite of SFS, in that the whole dataset deletes the features until the remaining features satisfy the stop criteria. The corresponding accelerated version is Generalised SBS. SFS ignores the correlations between features, and while SBS’s performance and robustness are preferable to those of SFS, the former needs more computational time. A single optimal combination of features is obtained by calculating and ranking the estimated value of each feature to obtain a combination that comprises the d preferential features. This method can only achieve a good combination of features when the estimated value of a single feature can be summed or multiplied. A random strategy is one in which the sub-features are totally randomly generated. In probability random feature selection, sub-features are chosen based on the given probability. Although the computational complexity of a random search strategy is still O(2^*N*^), it can drop to less than O(2^*N*^) if the maximum iteration is defined. Feature selection is essentially a combinatorial optimisation problem that can be tackled using non-global optimal target search methods and swarm intelligence random algorithms. Therefore, this strategy combines feature selection, with the simulated annealing algorithm^19^, or genetic algorithm (GA)^20^, or particle swarm optimisation (PSO) algorithm^21^ or the bootstrap approach^22, 23^. Intelligence algorithms always have multiple parameters that affect and determine whether a method can achieve optimal performance. Therefore, the performance of these intelligence algorithms directly affects the selection of optimal sub-features.

At present, the latter two strategies have not been shown to guarantee an optimal solution. However, feature selection based on swarm intelligence algorithms with random and reasonable heuristic searching strategies have been widely applied^11, 12^ to practical applications in finding second-best solutions with relatively fast computational speeds.

The evaluation standards for features can be grouped into filter^24^ and wrapper^25^ approaches. The wrapper approach uses the performance of machine learning algorithms as evaluation standards to estimate the selected features. In contrast, the filter approach is dependent without learning algorithms. Hence, compared with the filter approach, the wrapper approach is more complex but can achieve sub-features with better performance. A feature selection methods-based filter approach has a higher computational efficiency to evaluate the quality of features with certain metrics, such as distance^26^, information gain^27^, correlation^28^ and consistency^29^. The RELIEF^30^ series algorithms are commonly used for filter approach. A RELIEF algorithm aims to solve binary class datasets. First, it randomly selects m samples from the training dataset based on: the difference between each selected sample with its two nearest samples, respectively, in the same and different classes, to calculate correlations between each feature of the selected samples and each class; then, the average values of multiple selection as the weights of each feature; and finally, the algorithm obtains the correlation between each feature and class. Selecting the features with higher weights as selected feature combinations, RELIEFF^31^ is an extended version for solving multi-class and regression problems. It estimates the selected features as the closed samples’ identification abilities; that is, the samples with better feature combinations in the same classes are closed in the search space, and vice versa.

The wrapper approach in feature selection depends on the machine learning algorithm. It uses the selected sub-feature set to train the machine learning algorithm directly, then estimates the quality of the selected sub-feature set’s performance in testing the machine learning algorithm. The wrapper approach can achieve a significant solution when it combines machine learning (classification) and random strategy algorithms, which are mentioned in the previous section together. Previous researchers have combined GA and decision trees into classification models that select the optimal combination of features with the lowest error rate^32^; moreover, they have combined different classifiers – such as neural networks, Naive Bayes^11, 12^ and support vector machines^32^ – with random strategy algorithms, such as PSO and bat-inspired (BAT) algorithms^11^ to optimise the wrapper approach. Therefore, researchers are constantly trying to optimise machine learning and random strategy algorithms to enhance their computational efficiency and the quality of selected features. Given that the wrapper approach requires that these classifiers be constantly called and trained to verify and evaluate the performance of selected sub-feature sets, it takes more computation time than the filter approach. The wrapper approach offers higher accuracy, but when tackling high-dimensional datasets, the filter approach is more commonly used.

## Results

The classification results are assessed by different training and testing parts. We perform a strict 10-fold cross-validation^33, 34^ to test the corresponding performance of the current dataset classification model. The dataset is randomly subdivided into ten parts, based on averages, and each part takes a turn being the testing dataset with the other nine parts as training datasets in the repeated ten-times classifications. Accuracy and other performances of this cross validation process are then averaged from these ten classifications. To maintain the fairness of the experiment, because our proposed method, PSO, BPSO and WSA are random searching strategy algorithms, their experiments are also repeated ten times, and the final results are used as the mean value.

Tables 1 to 3 record the accuracy, dimension (%) and kappa statistics^35, 36^ of the selected sub-datasets with different methods. Tables 4 and 5 present the precision and recall values, respectively, to help us evaluate and compare these methods. Given the randomness of swarm intelligence algorithms, the results of this category in this time are the average values of their offsets (stand deviations) to verify and reflect the impartiality of our experiment.

**Table 1:** Accuracy of all datasets with different methods (best results highlighted in bold).

Accuracy	ALLAML	GLI_85	Prostate_GE	SMK_CAN_187	Colon	Leukemia
ELM	0.61	0.66	0.53	0.5	0.52	0.6
CHSAE	0.65	0.64	0.57	0.50	**0.71**	0.69
INFORGAE	0.68	0.67	0.61	0.50	0.63	0.72
RFAE	0.56	0.61	0.52	0.48	0.68	0.58
PSO	0.71 ± 0.03	0.69 ± 0.05	0.66 ± 0.04	0.53 ± 0.02	0.64 ± 0.06	0.68 ± 0.05
BPSO	0.70 ± 0.06	0.70 ± 0.06	0.7 ± 0.08	0.54 ± 0.04	0.66 ± 0.02	0.68 ± 0.05
WSA	0.72 ± 0.04	0.73 ± 0.04	0.66 ± 0.11	0.56 ± 0.04	0.66 ± 0.06	0.68 ± 0.05
EBWSA	**0.78 ± 0.0**8	**0.74 ± 0.03**	**0.81 ± 0.04**	**0.66 ± 0.06**	0.68 ± 0.04	**0.79 ± 0.06**

**Table 2:** Dimensions of all datasets with different methods.

Dimension	ALLAML	GLI_85	Prostate_GE	SMK_CAN_187	Colon	Leukemia
ELM	7130	22284	5967	19994	2001	7071
CHSAE	1026	192	1354	1867	28	1250
INFORGAE	2432	3562	2451	1727	220	1321
RFAE	5898	16780	4514	12525	1320	5569
PSO	3674.1 ± 1816.1	12456.5 ± 6205.8	2018.9 ± 1742.6	9505.2 ± 7589.8	1025.8 ± 687.3	2526.9 ± 2057.1
BPSO	3674.1 ± 566.5	12456.5 ± 5619.5	2018.9 ± 2258.6	9505.2 ± 5127.3	1025.8 ± 479.1	2526.9 ± 2339.6
WSA	3925.7 ± 3401.21	11174.3 ± 7988.38	2816.6 ± 2866.1	6406.4 ± 8237.1	1823.1 ± 368.3	5365.1 ± 2900.9
EBWSA	1098.4 ± 2179.4	8267.2 ± 5777.9	43.2 ± 59.4	25.3 ± 22.6	818.4 ± 554.6	972.5 ± 1554.1

**Table 3:** Kappa statistics for all datasets with different methods (best results highlighted in bold).

Kappa	ALLAML	GLI_85	Prostate_GE	SMK_CAN_187	Colon	Leukemia
ELM	0.16	0.17	0.12	−0.0174	−0.06	0.06
CHSAE	0.37	0.19	0.14	−0.0165	**0.39**	0.37
INFORGAE	0.39	0.24	0.22	−0.0149	0.23	0.43
RFAE	0.06	0.08	0.04	−0.0427	0.28	0.1
PSO	0.36 ± 0.07	0.21 ± 0.12	0.32 ± 0.08	0.07 ± 0.05	0.21 ± 0.14	0.30 ± 0.11
BPSO	0.33 ± 0.12	0.25 ± 0.15	0.39 ± 0.17	0.08 ± 0.07	0.26 ± 0.06	0.30 ± 0.10
WSA	0.38 ± 0.08	0.30 ± 0.10	0.32 ± 0.21	0.12 ± 0.09	0.26 ± 0.14	0.30 ± .012
EBWSA	**0.52 ± 0.08**	**0.34 ± 0.1**	**0.61 ± 0.21**	**0.32 ± 0.09**	0.29 ± 0.14	**0.53 ± 0.12**

**Table 4:** Precision of all datasets with different methods (best results highlighted in bold).

Precision	ALLAML	GLI_85	Prostate_GE	SMK_CAN_187	Colon	Leukemia
ELM	0.68	0.34	0.46	0.33	0.62	0.74
CHSAE	0.82	0.42	0.56	0.34	0.73	0.68
INFORGAE	0.62	**0.5**	0.66	0.37	0.65	0.7
RFAE	0.59	0.35	0.48	0.41	**0.78**	0.66
PSO	0.76 ± 0.04	0.34 ± 0.09	0.62 ± 0.06	0.41 ± 0.11	0.72 ± 0.07	0.77 ± 0.07
BPSO	0.74 ± 0.07	0.38 ± 0.12	0.65 ± 0.15	0.48 ± 0.10	0.72 ± 0.02	0.76 ± 0.08
WSA	0.78 ± 0.05	0.40 ± 0.09	0.64 ± 0.13	0.47 ± 0.14	0.75 ± 0.05	0.75 ± 0.05
EBWSA	**0.85 ± 0.10**	0.47 ± 0.11	**0.81 ± 0.07**	**0.65 ± 0.10**	**0.78 ± 0.04**	**0.83 ± 0.07**

**Table 5:** Recall of all datasets with different methods (best results highlighted in bold).

Recall	ALLAML	GLI_85	Prostate_GE	SMK_CAN_187	Colon	Leukemia
ELM	0.71	0.45	0.52	0.46	0.62	0.65
CHSAE	0.72	0.44	0.56	0.46	**0.8**	0.82
INFORGAE	0.81	0.46	0.58	0.47	0.74	0.84
RFAE	0.62	0.36	0.51	0.45	0.73	0.68
PSO	0.78 ± 0.03	0.51 ± 0.11	0.67 ± 0.05	0.53 ± 0.03	0.72 ± 0.06	0.75 ± 0.04
BPSO	0.75 ± 0.04	0.53 ± 0.12	0.70 ± 0.08	0.53 ± 0.05	0.74 ± 0.03	0.76 ± 0.04
WSA	0.80 ± 0.03	**0.60 ± 0.10**	0.66 ± 0.11	0.55 ± 0.04	0.74 ± 0.06	0.76 ± 0.05
EBWSA	**0.82 ± 0.05**	0.59 ± 0.08	**0.80 ± 0.04**	**0.65 ± 0.06**	0.75 ± 0.04	**0.84 ± 0.04**

**Figure 1:**
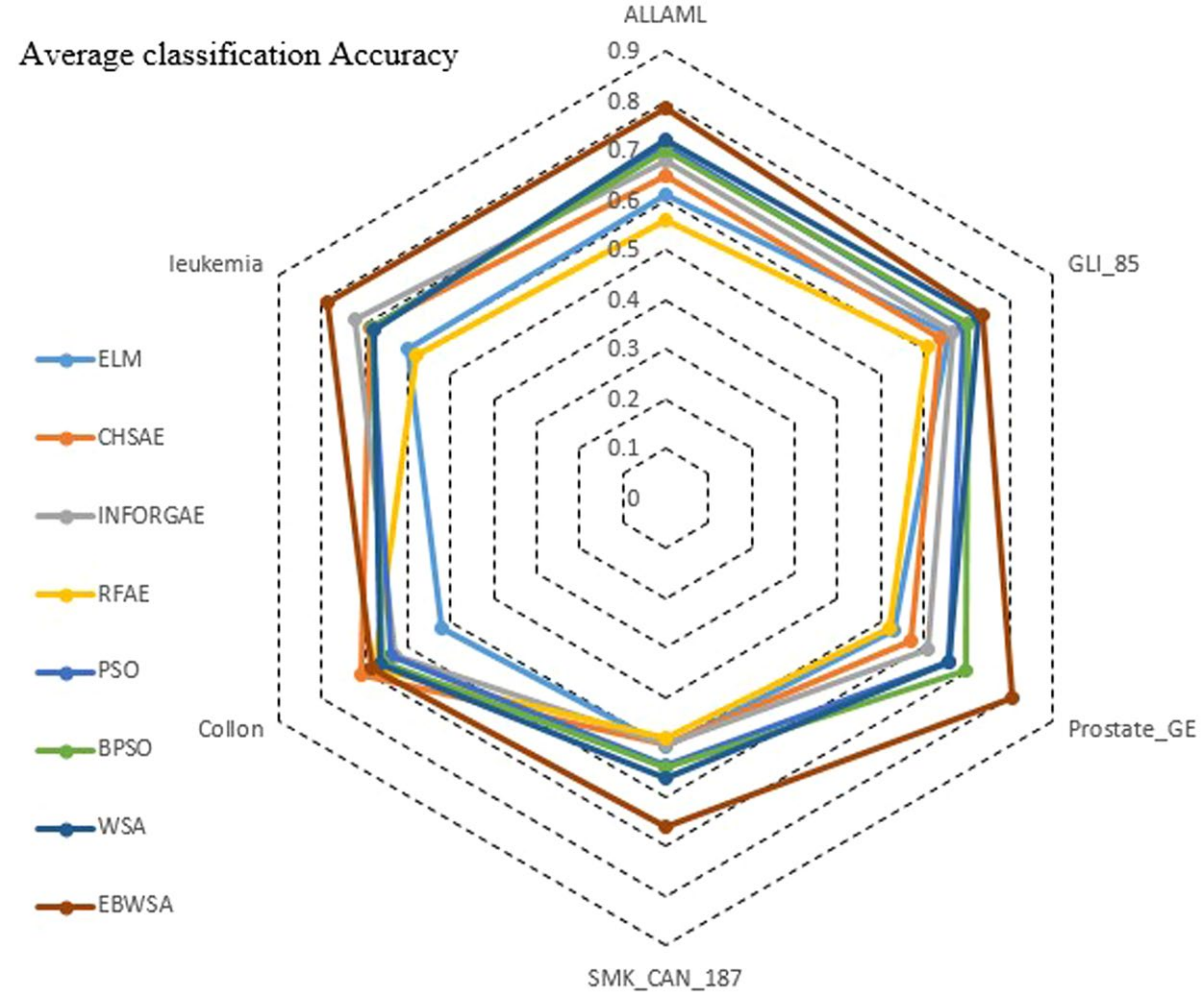
Average classification accuracy of all dataset.

**Figure 2:**
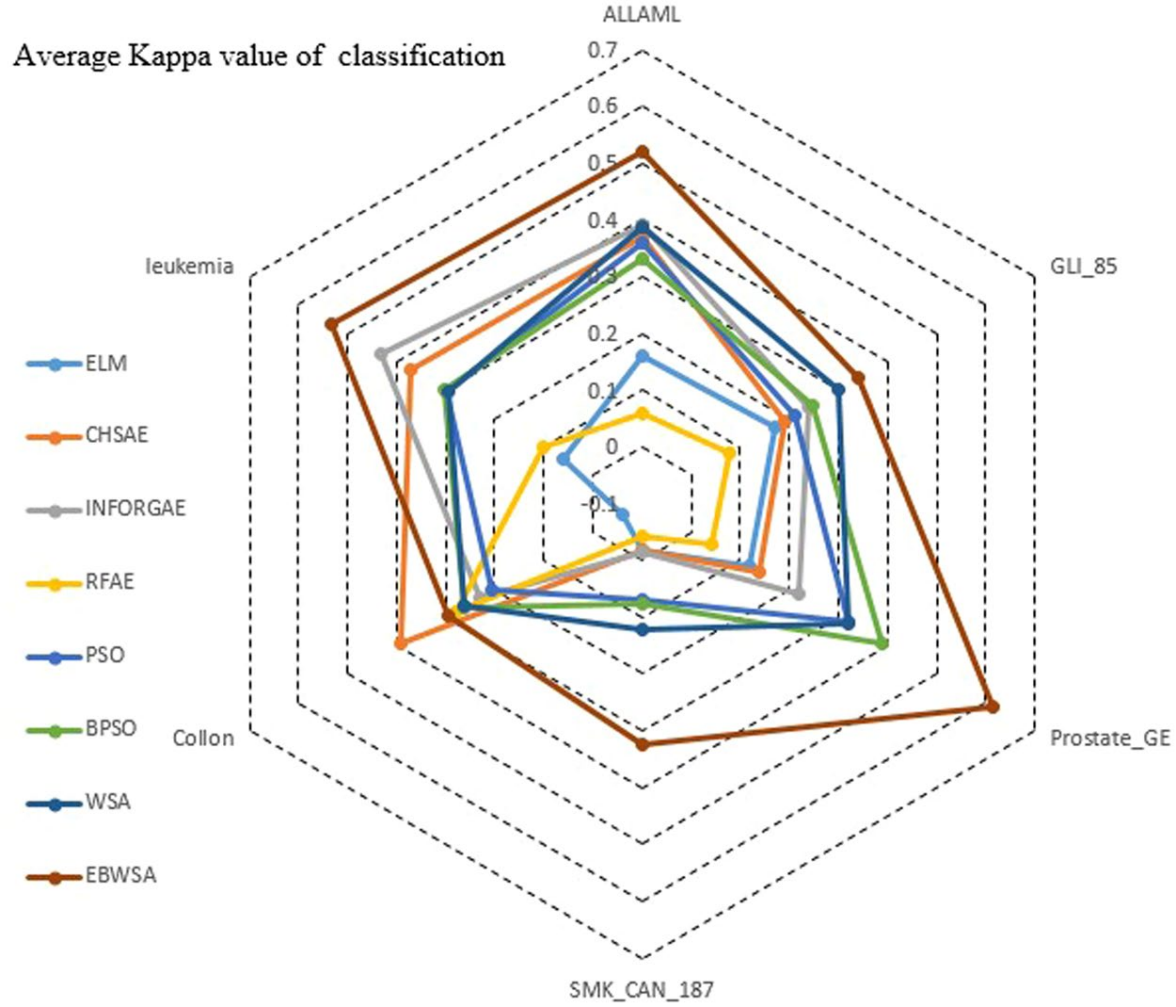
Average Kappa value of classification of all dataset.

The classification accuracy of the original all datasets for ELM is around 0.5 and 0.6. The first three methods with the heuristic and filter strategies improve the accuracy a little, whereas the RFAE obtain worse accuracy while processing high-dimensional datasets. The CHSAE is the best of the first three. It evaluates the chi-squared statistics of each feature with respect to the class. As mentioned in Section 2, heuristic searching strategies combined with filters can achieve some good effects, but random searching strategies based on a wrapper approach can obtain better feature sets with higher accuracy. Their worst algorithm (PSO) is still better than the CHSAE. Combining the results shown in Fig. 1 with the values in Table 1, and WSA, the binary version of PSO and BPSO are all better than PSO, which is a typical, effective swarm intelligence algorithm. However, it can be observed that WSA and BPSO do not increase the classification accuracy by much, whereas the features selected by the EBWSA exhibit more than a 10% increase in classification accuracy. The Kappa statistic is a value to measure the robustness of classification models^35^, with a bigger value indicating greater reliability. Table 3 and Fig. 2 illustrate that the robustness of the classification model for the original high-dimensional bioinformatics dataset is weak. After feature selection, the Kappa statistic of each classification model is enhanced for the bioinformatics dataset SMK_CAN_187 with filter methods. The EBWSA cycle in Fig. 2 is much bigger than the others, along with the point of the CHSAE for the Colon dataset.

**Figure 3:**
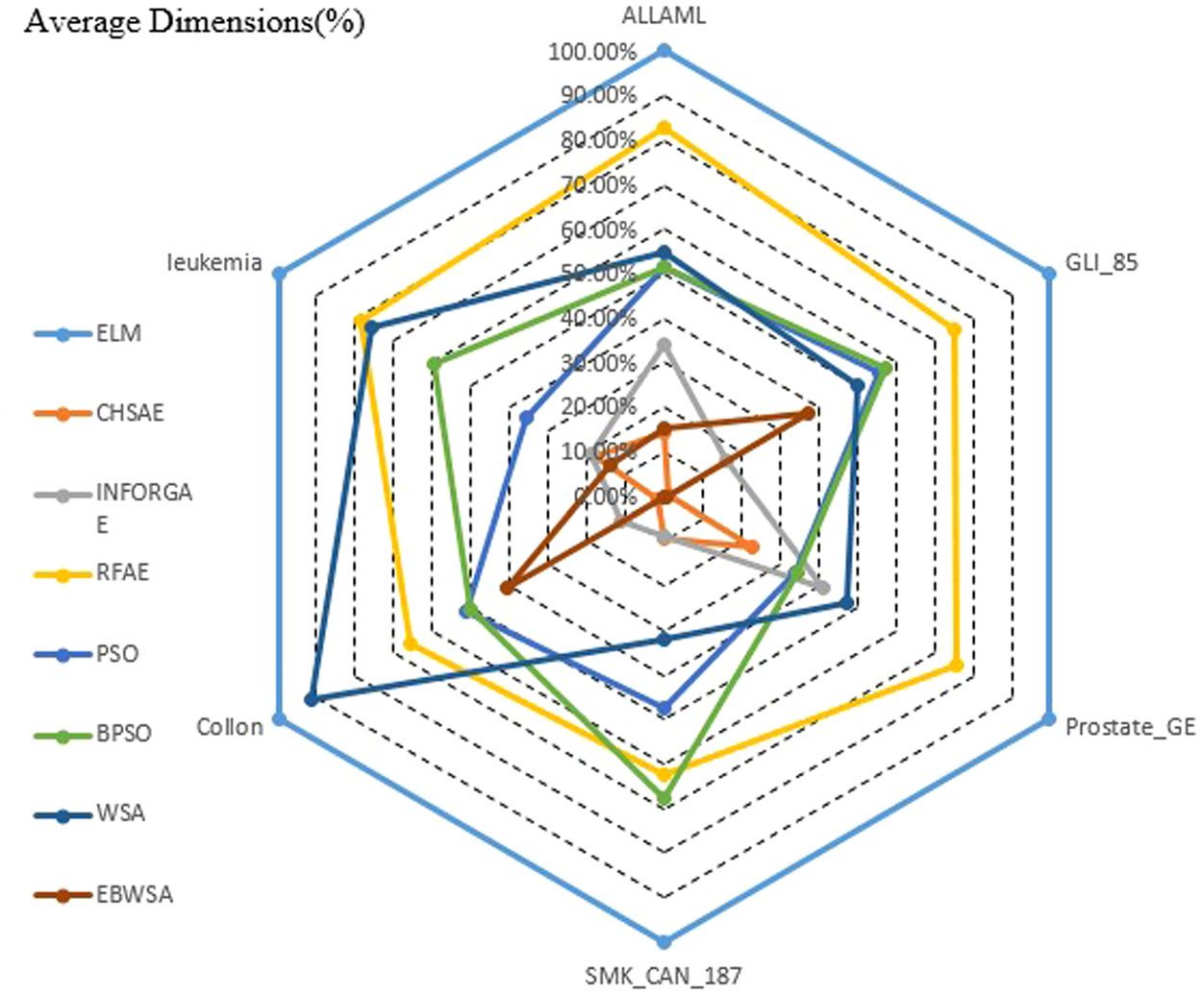
Average dimension (%) of all dataset.

Table 2 records the selected optimal features of each method and dataset, and Fig. 3 presents the percentage of the size of the original feature set that the selected optimal feature subset contains; that is, the length of the selected feature subset in the percentage of the maximum dimension of the original feature set^37^ or % dimension. It can be observed that the dimensions of experimental datasets are in the thousands and tens of thousands. Figure 3 intuitively reflects how the CHSAE and EBWSA selected smaller, more precise particles of feature sets, resulting in superior performances with respect to the filter and wrapper approaches. However, the lengths of selected features do not imply that more refined feature sets perform better, because INFORGAE was much worse than most of the other methods with longer lengths.

## Discussion

**Figure 4:**
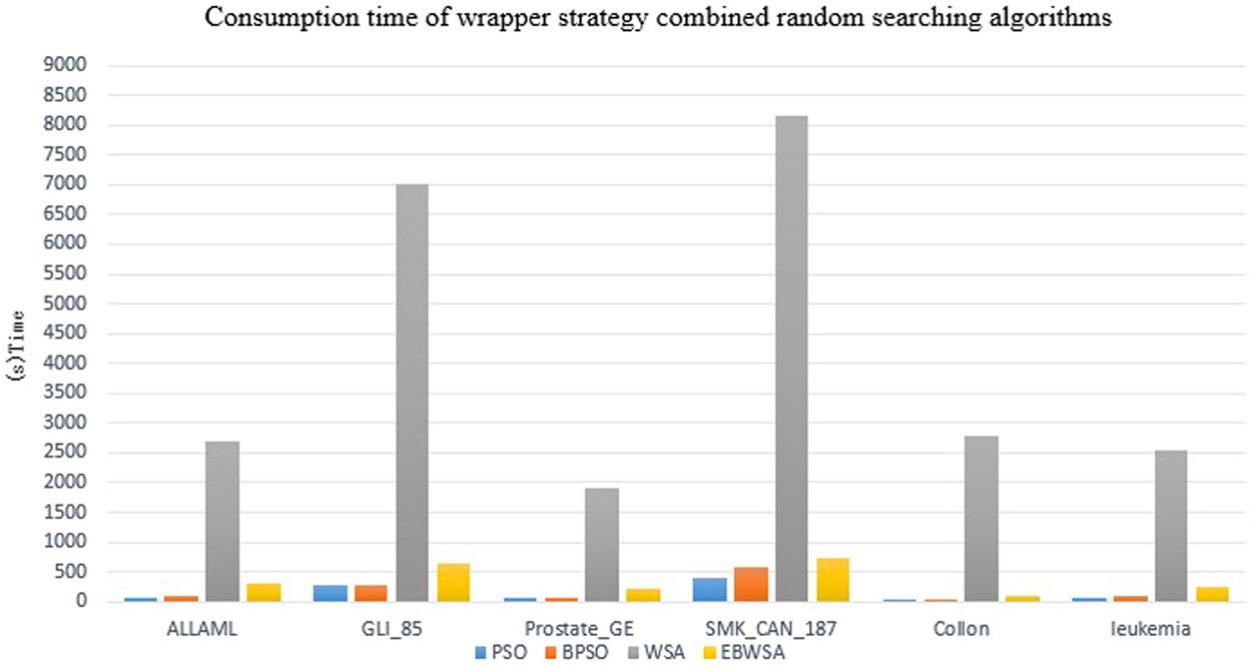
Consumption time by each swarm intelligence algorithm (i.e. PSO, BPSO, WSA, and EBWSA).

**Figure 5:**
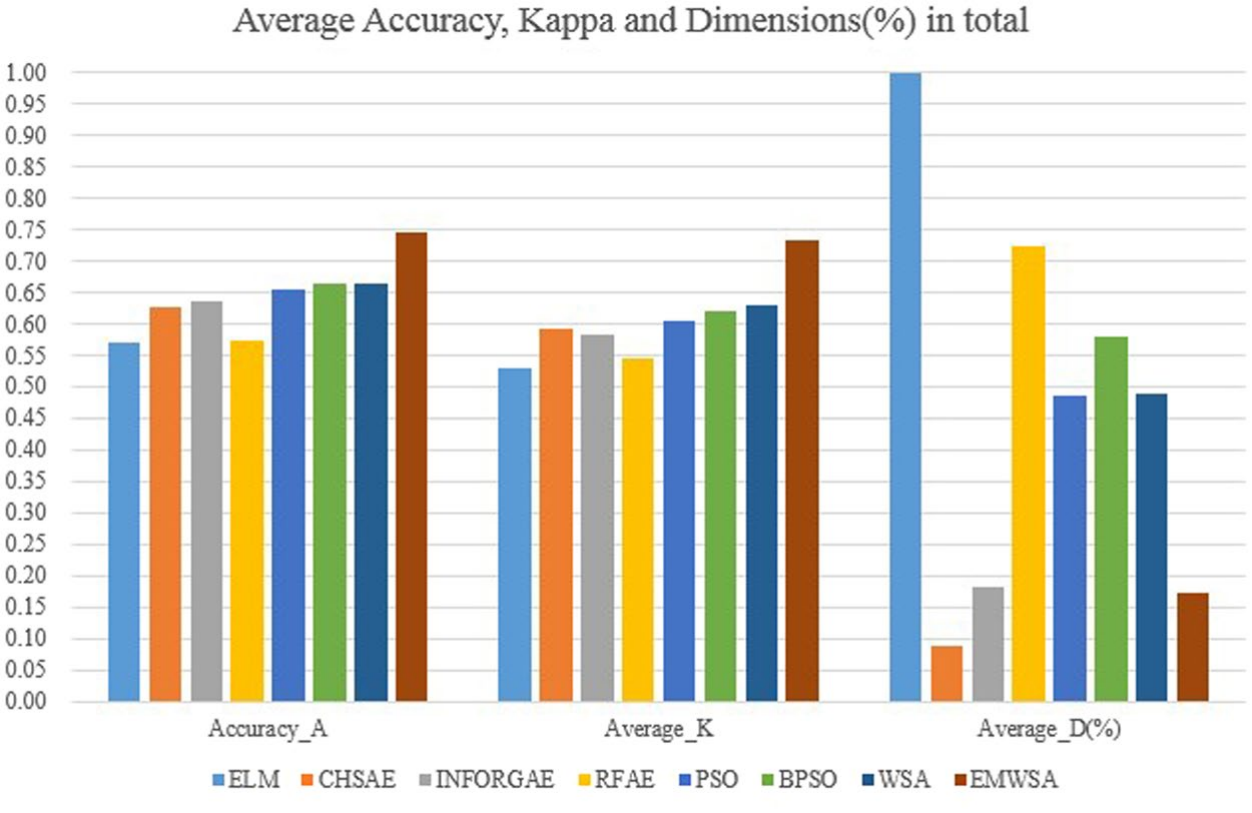
Average accuracy, Kappa and Dimensions (%) of the ELM, CHSAE, INFORGAE, RFAE, PSO, BPSO, WSA, and EMWSA methods.

It is indeed that filter methods are much faster than the wrapped random searching algorithms. The latter needs to call the classify tens of thousands of times. However, on the premise of the reasonable and acceptable time cost, wrapper is able to obtain better performances. Conventional WSA for feature selection is verified that it better than PSO and BAT with different classifiers in classification performance^11^. WSA’s shortcoming is that the large computation time because of its multi-leader and escape mechanism in a vast search space. Figure 4 displays the time cost of the proposed method with other three comprised swarm intelligence algorithms, the unit of time cost is the second. It is significantly observed that super time cost of WSA, and the extremely and effectively shorten the consumption time of EBWSA. Although EBWSA needs more time than PSO and BPSO, but they are sufficiently close to each other In addition, the experimental results indicate that it even enhances the computational up to 99.81% than WSA. Furthermore, EBWSA also could obtain the better the second-best optimal feature set with higher classification performance, it is displayed in Fig. 5. Figure 5 is the average accuracy, kappa and dimensions (%) in total of each method, besides RFAE, the results demonstrated the gradual growth from the left to the right. Wrapper is better than filter to have better selected features with higher performance of the classification model. Swarm intelligence algorithms is able to select more suitable length of selected features.

The conclusions are as follows. This paper proposes the EBWSA to optimise the feature selection for a high-dimensional bioinformatics dataset. Based on the WSA, the EBWSA selects a better second-best feature set with higher accuracy for classification within a more reasonable computation time. It uses the wrapper strategy that combines the EBWSA and ELM classification to implement the feature selection operation. The elitist strategy motivates stronger wolves to find better solutions in severe environments to accelerate population updates, and while the weaker wolves are assigned to some resources when the environment improves, the resources are executed according to variable weights for each wolf as their fitness values change. Based on their searching abilities, different wolves have different step sizes, and the memory function makes the search more effective by promoting the convergence of the population. Meanwhile, the binary approach diverts the feature selection problem to a similar function optimisation problem to obtain an optimal feature set with optimal classification accuracy and optimal length. The experimental results show that the wrapper approach is better than the filter approach in classifying selected features within a longer computing time. However, with an extremely high-dimensional dataset, the wrapper approach is more effective and useful than the filter approach within a reasonable time (when faced with tens of thousands of features, a few hundred seconds is needed to obtain a better solution). The EBWSA outperforms other conventional feature selection methods in classification accuracy by up to 29%, and it outperforms the previous WSA by up to 99.81% in computational time.

## Methods and Materials

### Elitist Binary Wolf Search Algorithm

**Figure 6:**
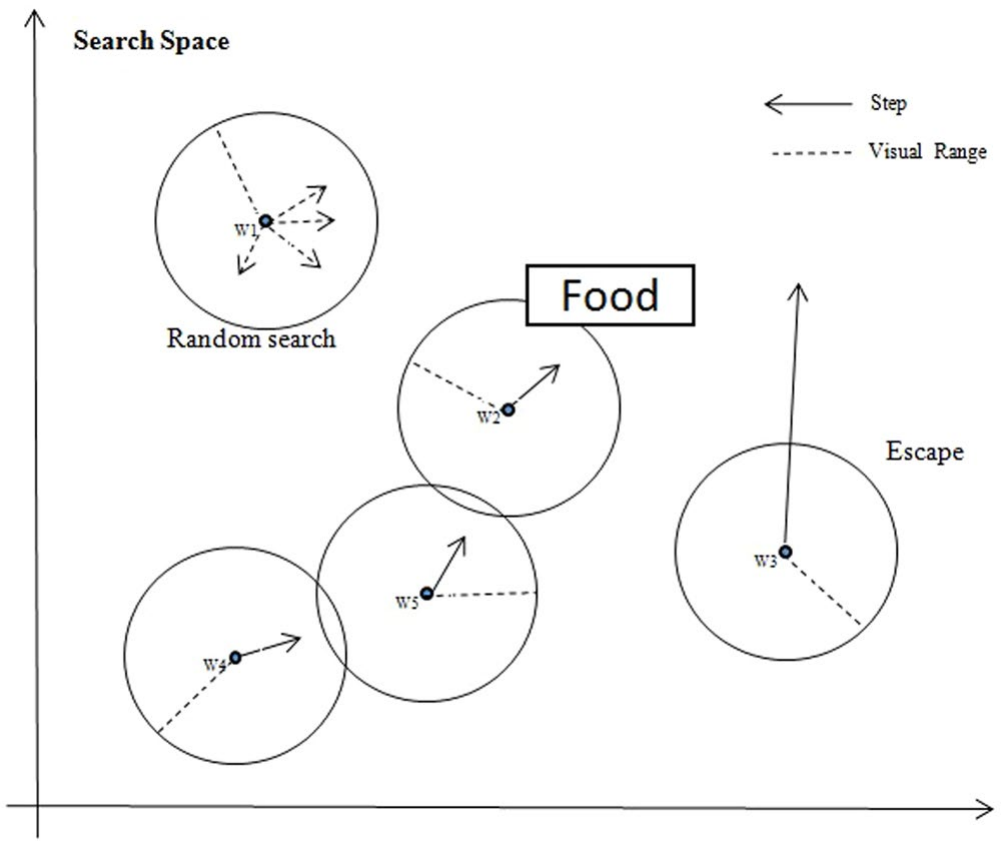
Hunting behaviors of WSA (based on an example of a population of five wolves in iteration).

As mentioned, the random strategy algorithm is an essential part of the wrapper model in feature selection. Different new algorithms are proposed to improve feature selection. The swarm intelligence algorithm, also called a bio-inspired algorithm, is a unique random strategy algorithm that exhibits significant performance (some examples include PSO^38^ and BAT^39^. As their names reflect, they are inspired by natural biological behaviour and use swarm intelligence to find an optimal solution. The WSA^10^ is a new swarm intelligence algorithm inspired by the hunting behaviour of wolves. However, it differs from the other bio-inspired algorithms because the behaviour in the WSA is assigned to each wolf rather than to a single leader, as in the traditional swarm intelligence algorithms. In other words, the WSA obtains an optimal solution by gathering multiple leaders, rather than by searching in a single direction. Figure 6 uses an example to show the hunting behaviour of WSA in 2-D.The original WSA observes three rules and follows the related steps to achieve the algorithm^37^:

Each wolf has a full-circle visual field in full rage with *v* as the radius. Here, the distances are calculated by Minkowski distance, as in Equation 1:

1 $$v\le d(x(i),x(c))={(\sum _{k=1}^{n}{|x(i,k)-x(c,k)|}^{\mu })}^{\frac{1}{\mu }},x(c)\in X,$$

where *x*(*i*) is the current position, *X* denotes all of the candidate neighbouring positions, *x*(*c*) is one of *X* and *μ* is the order of dimensional space. Each wolf moves towards its companions, who appear within its visual circle, at a step size that is usually smaller than its visual distance.

Equation 2 is the absorption coefficient, where *β*_*o*_ is the ultimate incentive and *r* is the distance between the food or the new position, and the wolf. Equation 2 is needed because the distance between the current wolf’s position and its companion’s position must be considered. The distance and attraction are inversely proportional, so the wolf is eager to move towards the position with the minimum distance.

2 $$\beta (r)={\beta }_{o}.$$

Given a different environment, the wolf may encounter its enemies. It will escape to a random position far from its current position and beyond its visual field. The following two equations are the movement formula:

3 $$x(i)=x(i)\,+\,{\beta }_{o}{e}^{-{r}^{2}}(x(j)-x(i))+escape(),$$

and

4 $$if\,moving=\{\begin{array}{c}x(i)=\,x(i)+{s}^{\ast }rand()Prey\\ x(i)=x(i)+{s}^{\ast }escape()Escape\end{array},$$

where the escape () function obtains a random position to jump to with a minimum length constraint, *x*(*j*) is the peer with a better position and better fitness than *x*(*i*) and s and *rand*() in equation 4 are the step size and a rand value within −1 and 1, respectively. Actually, the step size of the WSA is equal to the velocity of PSO. An escape machine effectively reduces the population until it falls to the local optimum.

The WSA outperforms the other swarm intelligence algorithms in accuracy of feature selection when used with the wrapper strategy^35, 37^. However, the multi-leader searching strategy and escape mechanism result in the need for better performance from selected features with a higher time cost. In a vast search space, a fixed step size limits wolves’ visual fields and movement speeds. The population of wolves converges slowly towards the optimal solution, and as mentioned, the wrapper strategy typically needs more computation time. Therefore, we proposed the Elitist Binary Wolf Search Algorithm (EBWSA) to improve the performance and reduce the time cost of the WSA for feature selection.

**Figure 7:**
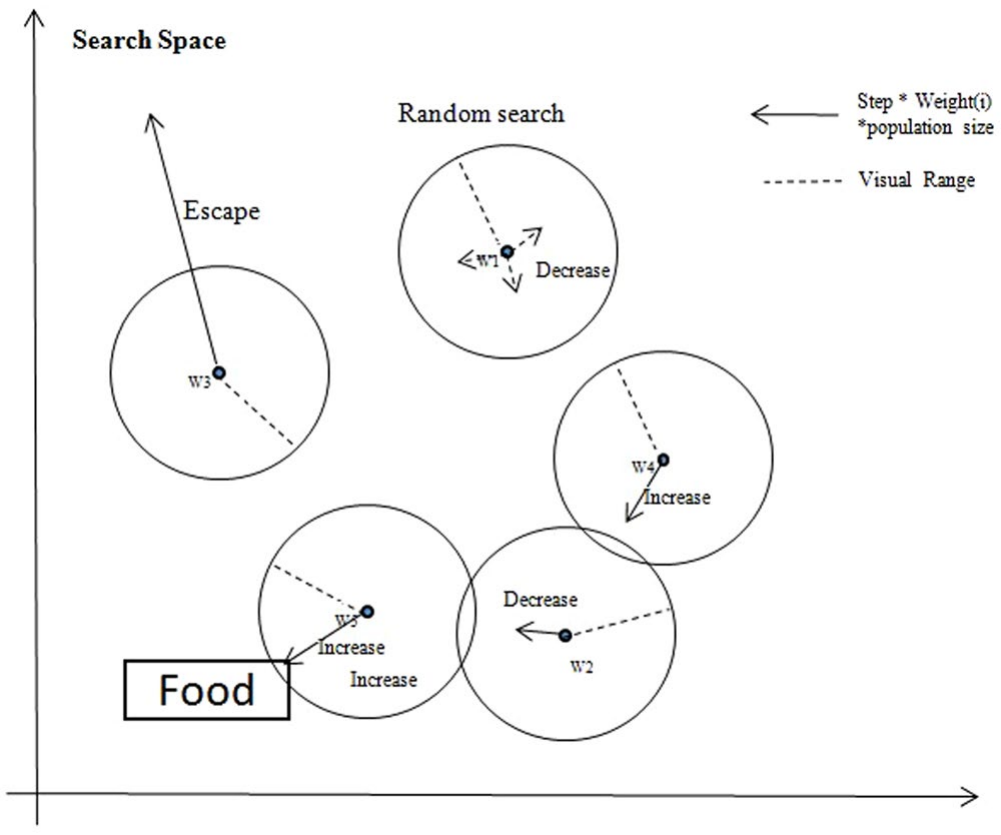
Hunting behaviors of EBWSA (based on an example of a pack of five wolves in an iteration).

The EBWSA, which is based on the WSA^10^, uses the weight of each wolf while searching, to determine the step size dynamics for their fitness values in the current iteration, to update their own weights 44. In initiation, each wolf is treated as a weak searcher. During the process of searching, the stronger wolves who find the better results with weights that are less than the half of the total gain more weight and become Elitist wolves. In contrast, under this mechanism, the wolves with poor ability weaken, as their Elitist counterparts take more than half of the total weight. If the Elitist wolves take less than half the total weight, it means that the living environment is poor, which will motivate them to gain more resources. When the living environment improves, the weak wolves gain resources to balance the whole population. This simulates Darwinian evolution^40^; specifically, natural selection and survival of the fittest. To avoid throwing the whole population out-of-balance, a weak wolf will be eliminated and reborn as a stronger wolf while its weight touches the elimination and reborn threshold for searching, the normalized operation of weights will redistribute the weights to each wolf. Meanwhile, if any wolf’s weight is equal to or more than half of the whole, its weight will be reset and the weights of the population will be redistributed. Inspired by the Eidetic WSA^9^, the EBWSA also has a memory function to avoid repetition and promote efficient searching. This function records the worst position of the wolf at each iteration, so that wolves in subsequent iterations will keep away from the previous worst positions. To reduce the time cost, several earlier records of the worst memory position will be forgotten once the default memory length is full. This operation makes the whole population more intelligent to fulfil the imitated population in nature with the memorising–forgetting mechanism. Figure 7 in the diagram below demonstrates the 2-D hunting behaviour of a pack of five wolves with their weights being adjusted in an iteration according to EBWSA.

As the example was shown in Fig. 7. In the last iteration, if the total weight of better wolves is smaller than 0.5, the better wolf means its fitness is better than its previous iteration. Determine the current living environment is worse. The better wolf’s weight will be increased in the next iteration. In this diagram, *w*4 and *w*5 are better wolves in worse living environment in current iteration. On the contrary, if the total weight of the better wolves is bigger than 0.5 in the last iteration, the worse wolves will get some weights from the better wolves to increase their weights in better living environment, at this time, *w*1 and *w*2 are better wolves in current iteration. In addition, if total weight of better wolves is equal to 0.5, the wolves will keep their own weight. It needs to be noted that, if the weight of *w*4 or *w*5 bigger than 0.5, the whole weight will be re-given; else if the weight of *w*1 and *w*2 smaller than one percent of the initial weight, the whole weight will be re-assigned and these two positions will be reborn. The following are the weight variation steps:

1. The total weight is 1, each wolf’s weight is *1*/*N (W*_*i*_ *)* in the initial phase and *N* is the size of the population.
2. For *m* = 1,…, *M* (*M* is the maximum iteration time)

- get γ,
  5 $$\gamma =\,\sum _{0}^{t}({W}_{m,i})[if\,fitnes{s}_{m,i} > \,fitnes{s}_{m-1,i}\,]$$
  where *t* is the number of better wolves and *γ* is the sum of the better wolves’ weights.
- Choose ${\sigma }_{m}\,\,$to measure the living condition of the population:
  6 $${\sigma }_{m}=0.5\,\ast \,ln(\frac{1-\gamma }{\gamma }),$$
  when *γ* < 0.5, *σ*_*m*_ > 0. If *γ* decreases, *σ*_*m*_ increases. This means that if the current population of Elitist wolves takes less than half of the total resources, then the living environment is worse. If *γ* is bigger than 0.5, it indicates that the environment is good.
- Update the distribution of weights for the wolves. If *γ* is smaller than 0.5, then *σ*_*m*_ has a positive value. If ${e}^{{\sigma }_{m}}$ is bigger than 1, then ${W}_{m+1,i}$ will increase in the next iteration. In other words, in a poor living environment, the weights of these wolves must be increased to motivate them. In contrast, the weaker wolves will have some weights that equal the population in a better environment. When the population is weak overall, the Elitist wolves will gain increasing rewards. When the population is strong overall, the weaker wolves need help.
  7 $${W}_{m+1,i}={W}_{m,i}\ast \{\begin{array}{c}{e}^{{\sigma }_{m}}\,if\,\,fitnes{s}_{m,i}is\,better\,than\,fitnes{s}_{m-1,i}\\ {e}^{-{\sigma }_{m}}\,if\,fitnes{s}_{m,i}is\,worse\,than\,fitnes{s}_{m-1,i},\end{array}$$
- Normalise the weights so that the resources in the environment are constant.

8 $${W}_{m+1,i}=\frac{{W}_{m+1,i}}{{\sum }_{0}^{N}{W}_{m+1}}.$$

The updated formulas in equations (3) and (4) become equations (9) and (10). In equation 9, each wolf needs to multiply the corresponding weights to update its position. The dynamic weight changes the fixed value of the step size to realise the Elitist wolf, who is able to go further in its searching. The total value of the weights is normalised in equation (8). Thus, ${w}_{i,j}$ is less than 1, and ${w}_{i,j}\ast N$ is a value floating around 1 to change the step size.

9 $$x(i)=x(i)+\,{\beta }_{o}{e}^{-{r}^{2}}(x(j)-x(i))\ast {w}_{i,j}\ast N+escape(),$$

10 $$if\,moving=\{\begin{array}{c}x(i)=x(i)+s\ast {w}_{i,j}\ast N\ast rand()Prey\\ x(i)=x(i)+s\ast {w}_{i,j}\ast N\ast escape()Escape\end{array},$$

The EBWSA optimises the feature selection process into a binary optimisation problem. The numbers of features stand for the dimensions and positions of each feature. This means that in the EBWSA’a feature selection, the position of each individual particle can be given in binary form (0 or 1), which adequately reflects the straightforward ‘yes/no’ choice of whether a feature should be selected. The scope of a position is from −0.5 to 1.5. Then, equation (9) is used to calculate the binary value of the position.

11 $${X}_{i}^{j}=\{\begin{array}{c}round({x}_{i}^{j})=1\quad 0.5\le {x}_{i}^{j} < 1.5\\ \,=0\quad \quad \quad \quad otherwise\end{array},$$

where ${x}_{i}^{j}$ denotes the particle *x*(*i*) in position(dimension) *j* and the round() function calculates the binary value ${X}_{i}^{j}$ of the corresponding position to achieve the binary optimisation operation.

**Figure 8:**
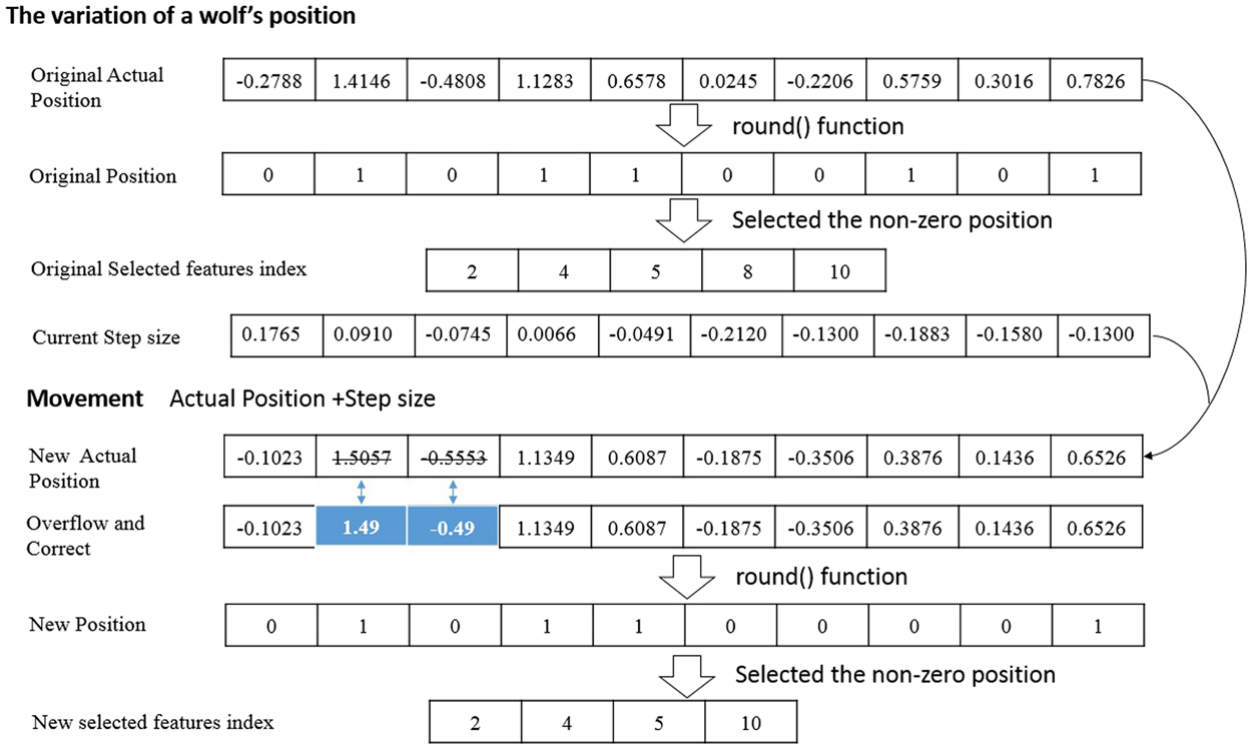
The variation of a wolf’s position during iteration.

**Figure 9:**
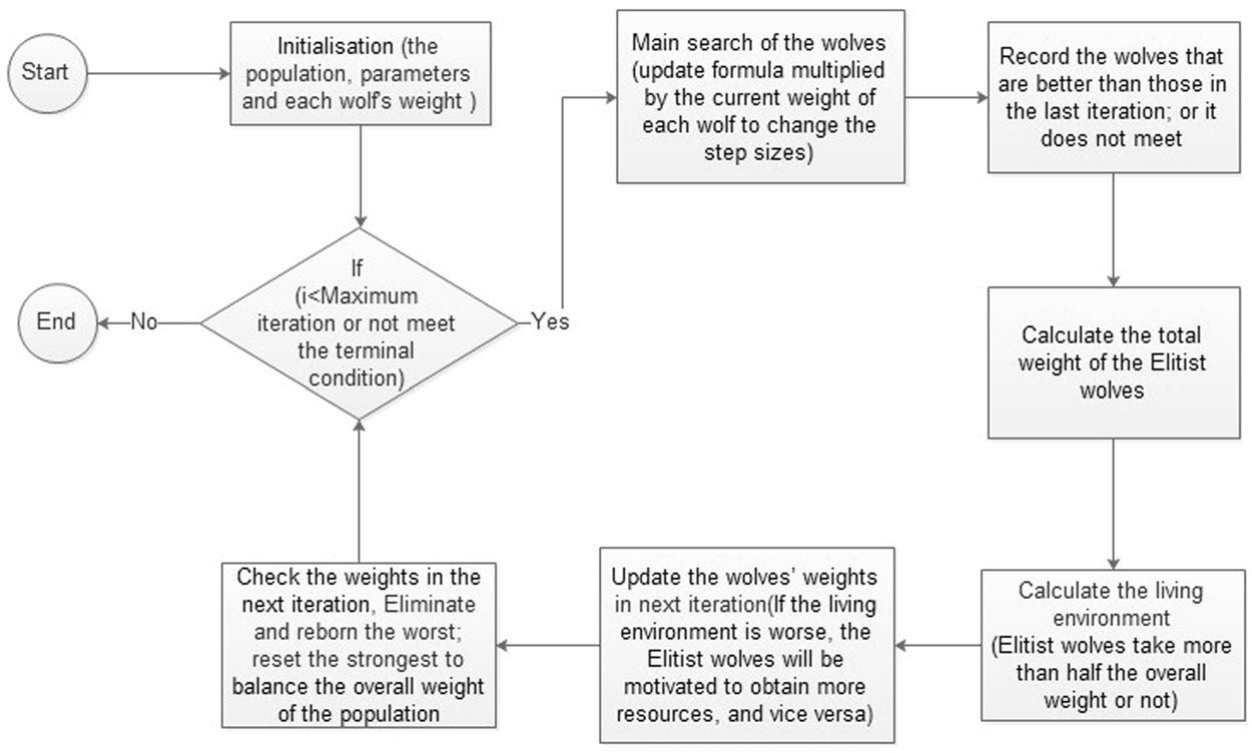
Flow chart of the elitist process of WSA.

Figure 8 presents a wolf’s movement in an iteration of a binary strategy. The EBWSA’s feature selection can be regarded as a high-dimensional function optimisation problem, wherein the values of the independent variables are 0 or 1. In addition, values of 0 and 1 can also be given to dependent variables calculated by the rounding function, whose independent variables can be assigned from −0.49 to 1.49. The step size of each position is a very small value in a fixed range. At the beginning of Section 2, we described the classical definition of feature selection as selecting a sub-dataset *d* with *f* features from the primary dataset *D* with *F* features, *f* ≤ *F*, where d has the optimal performance in all of the sub-datasets with f features from the primary dataset^6^. Thus, we know that the value of *f* is a defined value in this definition, and while it should be a variable, that means that algorithms should find the optimal length with an optimal combination. The EBWSA repairs this problem to obtain the optimal feature set using a similar method of function optimisation. Figure 9 illustrates the weighted EBWSA process, and we present pseudo EBWSA code.

**Table:** 

EBWSA Pseudo code
Objective function *f*(*x*), *x* = (*x*_1_, *x*_2_,..*x*_*d*_)
Initialize the population of wolves *x*_*i*_(*i* = 1,2,.., *M*)
Define and initialize parameters:
The memory length
*T* = Maximum iterations
*r* = radius of the visual range
*s* = basic step size of each wolf
*W= (w*_*1,1*_, *w*_*2,1*_, *w*_*3,1*_ *,…*, *w*_*i,j*_ *,…*, *w*_*M,T*_ *)*
// different weights of different wolves in different iteration, *i∈M* and *j∈T*
*w*_*1*,*1*_ *=* *w*_*2*,*1*_ *=* *w*_*3,1*_, *=* , *w*_*M*,*1*_ *=* *1*/*M*
*p*_*a*_= a user-defined threshold between 0 and 1
**1**. Initial the fitness of wolves with their own weights.
**2. While(** *j*<*T* and stopping criteria is not satisfied)
**3**. ppfitness = fitness; //store the previous fitness in ppfitness
**4. FOR** *i =*1: *W // for each wolf*
**5**. Prey_new_food_initiatively × *w*_*i,j*_
**6**. Generate_new_location// check the location is in the memory or not, if yes, repeat generate new location
**7. IF** the distance of two wolves are less than r and one is better than the other one
**8**. the better one will attract the other one to come over
9. ELSE IF
**10**. Prey_new_food_initiatively × *w*_*i,j*_
11. END IF
**12**. Generate_new_location
**13. IF** (*rand* *>* *p*_*a*_)
**14**. This wolf will escape to a new position // check the location is in the memory or not,
if yes, repeat generate new location
15. END IF
**16. IF** current fitness of *x*_*i*_ stronger than the previous
**17**. Recorded *x*_*i*_ in a set of *IDw* // re-order the stronger wolves
18. END IF
19. END FOR
**20**. *γ* is the sum of the wolves’ weights in *IDw*
**21. IF** *γ!* *=* *0*
***22*** *. σ*(*j*) *=* (1/2) × (*log*(*γ*/(1*- γ*)));
23. ELSE
**24**. *σ*(*j*) *=* (*1*/*2*) × (*log*(1));
25. END IF
// Update the weight
**26. FOR** *i* *=* 1: *W*
**27. IF** the wolves in *IDw*
**28**. *W*_*i*, *j+1*_ *=* *W*_*i,j*_ × *exp*(*σ*(*j*)) //enhance the weight of stronger Wolves in next iteration
29. Else
**30**. *W*_*i*, *j+1*_ *= W*_*i,j*_ × *exp*(*-σ*(*j*)) //reduce the weight of weaker wolves in next iteration
31. END IF
32. END FOR
**33**. Normalization processing *W*_(1*,…M*), *j+*1_
**34. FOR** *i* *=* 1: *W*
**35. IF** *W*_*i*, *j+*1_ *<*1/(*100* × *M*)
***36*** *. Reset (reborn) this wolf* // check the location is in the memory or not, if yes, repeat generate new location
**37**. *W*_*i*, *j+*1_ *=* 1/*M //* Re initialize this wolves ‘weight
***38*** *.* **ELSE IF** *W*_*i*, *j+*1_ *>* 1/2
**39**. *W*_*i*, *j+*1_ *=* *W*_*i*, *j+*1_ × *rand*(*0,1*) *//* Re give this wolves’ weight
40. END IF
41. END FOR
**42**. Normalization processing *W*_(1*,…M*), *j+*×_
**43**. Check the memory is full or not // if it is full, it will delete defined number of earlier positions//
**44**. Recorded the worst position in this iteration // check the location is in the memory or not
45. END WHILE

### The comments after the symbols “//” denotes explanatory information

The function Generate_new_location() calls for a classifier to calculate the accuracy of the classification model and return it as the fitness. What the EBWSA gathers is implemented using the above steps and codes to select the optimal feature set. In our experiment, we used classification accuracy as the evaluation metric to estimate the quality of the selected features. Higher classification accuracy signified a better combination of features, and vice versa.

**Figure 10:**
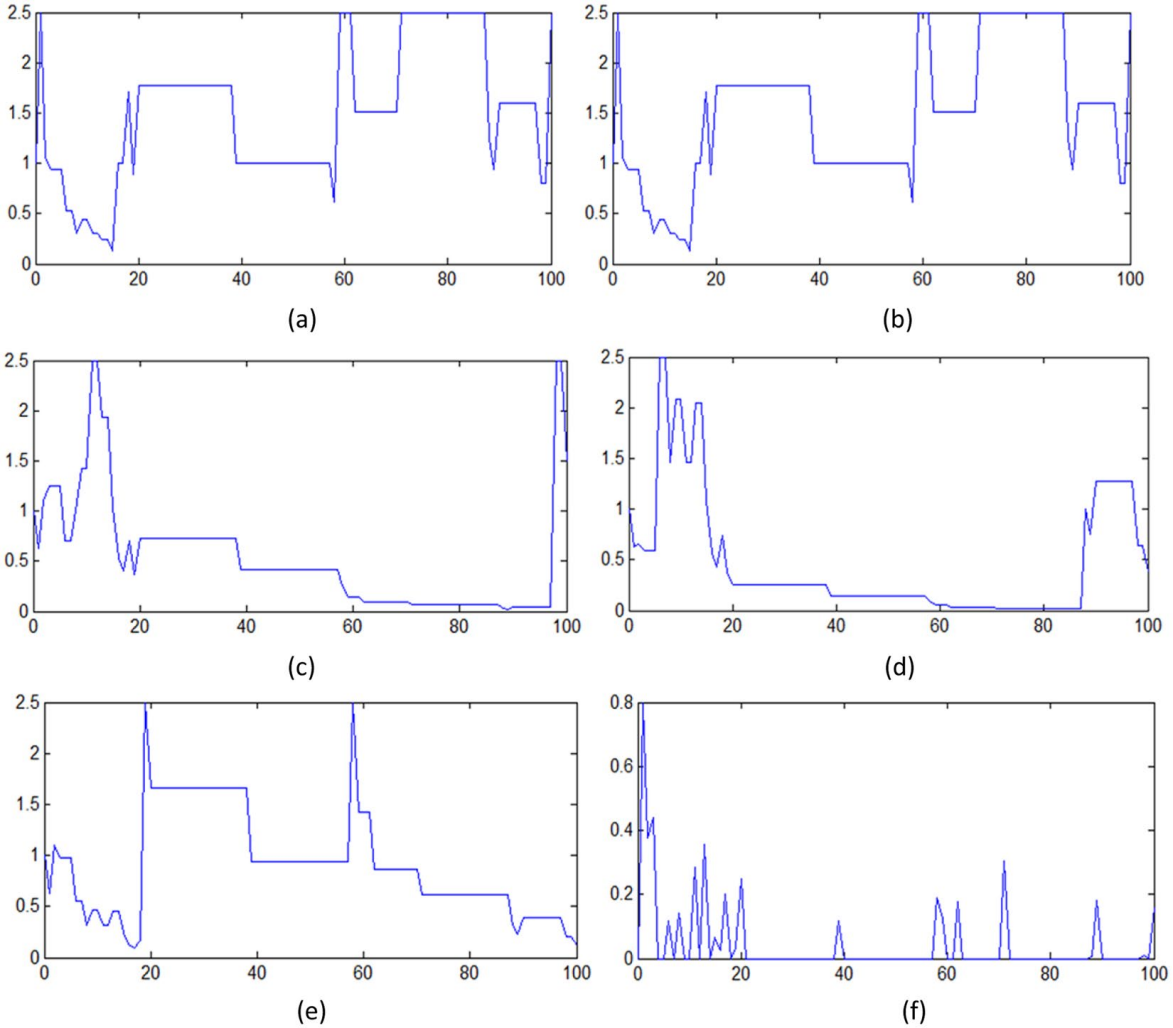
An example: variations of each wolf’s (weight × population) for a population of 5 within a maximum of 100 iterations in the sub-figures (**a–e**) and varations of living environments is showed in last sub-figure (**d**).

Figure 10 is an example of the EBWSA with 5 wolves and 100 iterations for the feature selection of the dataset Prostate_Ge – a high-dimensional bioinformatics dataset introduced in the next section. The first subfigure represents the wolves’ survival environment, which expresses the total weight of the Elitist wolves, whose fitness values are better than those of the wolves in the previous iteration. If this value is smaller than 0.5, the survival environment is considered to be bad, and the Elitist wolves will get more weight from their weaker counterparts. Because the search spaces of high-dimensional datasets are large, and the populations are small, most of the wolves have a difficult time finding a better solution. The next five subfigures describe the variations in each wolf’s (weight × population). If the weight is smaller than 1, then weight × population takes a value bigger or smaller than 1 to change the step size of each wolf. Therefore, these five subfigures indirectly present the step size changes of each wolf in 100 iterations.

### Dataset benchmarks

The six binary class bioinformatics datasets in Table 6 are used to test the effectiveness of the proposed method, and to compare the algorithms. They are biological data downloaded from the Arizona State University website^41^. It is observed that these are high-dimensional bioinformatics datasets with tens of thousands of features, besides datasets Colon and Leukemia. Such dimensions are commonly seen in biological or bioinformatics datasets.

**Table 6:** Bioinformatics datasets used in experiments.

**Data Set**	**#Instances**	**#Features**
ALLAML	72	7129
GLI_85	85	22283
Colon	62	2000
Prostate_GE	102	5966
SMK_CAN_187	187	19993
Leukemia	72	7070

### Comparison algorithms

In addition to the proposed methods, six algorithms are compared, three that use heuristic and filter strategies and three that use random and wrapper strategies. Classification accuracy is the evaluation metric for the selected features in our experiment. Hence, the first comparison is of the basic classifier extreme learning machine (ELM)^42^, which classifies the original high-dimensional datasets, and a traditional single hidden layer feed-forward neural network (SLFN) ELM that promotes the computational time cost under the premise that it guarantees learning accuracy. It is a network structure composed of an input layer, a hidden layer and an output layer. The hidden layer completely links the input and output layers. The whole learning process can be briefly divided into the following parts. First, determine the number of neurons in the hidden layer, then randomly set the threshold of neurons in the hidden layer and the connection weights between the input and hidden layers. Second, select an activation function to calculate the output matrix from the neurons in the hidden layer. Finally, calculate the output weights. Besides the ELM’s fast computational speed, simple parameters, strong generalisation ability and simple, quick construction of the SLFN make it ideal for use as the basic classifier in our experiment.

ELMs also classify datasets with selected features using different feature selection methods, and offer their classification accuracy for comparison. The first three approaches are chi-squared attribute evaluation (CHSAE), information gain attribute evaluation (INFORGAE) and RELIEFF attribute evaluation (RFAE) from the Waikato Environment for Knowledge Analysis^43^. CHSAE evaluates the worth of an attribute by computing the value of the chi-squared statistic with respect to the class, INFORGAE does so by measuring the information gain with respect to the class and RFAE does so by repeatedly sampling an instance and considering the value of the given attribute for the nearest instance of the same and different class. It can operate on both discrete and continuous class data. As mentioned before, these filter approaches rank attributes as the measured value. Thus, we retain and collect the features whose values are worth more than 0. The other three feature selection methods are separately wrapped PSO, binary PSO and a preliminary version of WSA with an ELM classifier to perform the feature selection operation and discover the feature combinations with the optimal accuracy of classification. The swarm intelligence iterative methods and ELM are programmed by Matlab 2014b with a population of 15 and a maximum of 100 iterations, inertia weight is 0.8. The computing platform for the entire experiment is CPU: E5-1650 V2 @ 3.50 GHz, RAM: 32 GB.
